# Serotype-specific tropism of adeno-associated viruses in dorsal meningeal lymphatic vessels via intra-cisterna magna delivery

**DOI:** 10.3389/fimmu.2026.1768041

**Published:** 2026-03-16

**Authors:** Bingxuan Ren, Xuhang Li, Xinru Lin, Wen Zhao, Kaixia Yang, Yuna Zhang, Wu Zheng

**Affiliations:** 1 Department of Neurology, The Affiliated Lihuili Hospital of Ningbo University, Ningbo University, Ningbo, Zhejiang, China; 2 Department of Operating Room, The Affiliated Lihuili Hospital of Ningbo University, Ningbo University, Ningbo, Zhejiang, China

**Keywords:** central nervous system, cerebrospinal fluid, delivery strategy, intra-cisterna magna, meningeal lymphatic vessels, recombinant adeno-associated virus

## Abstract

**Background:**

Meningeal lymphatic vessels (mLVs) in the brain acts as the regulator that eliminates harmful macromolecules in post-cerebrospinal fluid (CSF) and facilitates immune cell migration to cervical lymph nodes. However, it still poses challenges on the investigation of separated mLVs biological functions because of its unique anatomical position on the dorsal skull. Studies have been revealed that the recombinant adeno-associated virus (rAAV)2/1 serotype can be recognized as delivery vehicles targeting mLVs, which further can be exploited to evaluate mLVs biological functions. Nevertheless, the characteristics for infection of rAAV2/1 needs more investigations.

**Methods:**

In the present study, 8-week-old male C57BL/6 mice were selected, and rAAVs of different serotypes were injected either into the cisterna magna filled with CSF or via lateral ventricle injection, to compare the specificity of distinct rAAV serotypes for infecting mLVs.

**Results:**

Through intracisternal magna delivery, rAAV2/1-CMV-EGFP exhibited selective mLVs targeting with sustained fluorescence intensity. Additionally, rAAV2/5-CMV-EGFP, rAAV2/8-CMV-EGFP, rAAV2/9-CMV-EGFP, rAAV2/BR1-CMV-EGFP, and rAAV2/PHPeB-CMV-EGFP also showed the same trend. Significantly, rAAV2/9-CMV-EGFP infectivity via the injection in medullary cisterna induces EGFP expression in mLVs that is dose-dependent. However, at higher viral doses (≥1×10^9^ vg/mouse), expression reaches a plateau and maintains stability for 6 months, suggesting a saturation threshold of transduction efficiency.

**Conclusion:**

This study revealed the characteristics of diverse rAAV serotypes in mLVs research, suggesting that rAAVs can be regarded as a powerful and safe gene therapy tool for unveiling the role of mLVs under neuroinflammatory conditions or central nervous system (CNS) disorders.

## 1 Introduction

For decades, it has been widely accepted that the brain uniquely lacks a conventional lymphatic system, distinguishing it from other tissues in the body (1–3). Early evidence emerged in 1869 when tracers injected into the subarachnoid space were later detected in cervical lymph nodes (4), suggesting a drainage pathway connecting the brain to peripheral lymphoid tissues. However, the method of cerebral lymphatic drainage remained a subject of controversy, prompting the question: why is such a vital defense system absent in humans and animals?

A pivotal discovery in 2015 identified functional lymphatic vessels within the dural sinuses (5), providing structural evidence for routes that direct cerebrospinal fluid (CSF) and immune cells toward cervical lymph nodes. Subsequent studies have since linked meningeal lymphatic vessels (mLVs) to numerous central nervous system (CNS) disorders (6–9). For instance, in multiple sclerosis (MS), mLVs are part of the multifocal neuroinflammatory pathology and serve as a key delivery pathway for targeted therapy, enabling direct brain access and enhanced treatment efficacy (6). Impaired mLVs function disrupts CSF and interstitial fluid homeostasis, contributing to impaired hematoma clearance, hydrocephalus development, and subsequent neurological damage. Specifically, c-Src kinase is significantly activated following intraventricular hemorrhage (IVH), leading to phosphorylation of β-dystroglycan (β-DG) and consequent depolarization of aquaporin-4 (AQP4), thereby promoting the development and progression of hydrocephalus (7, 8). Conversely, restoring or enhancing mLVs function can promote drainage via the deep cervical lymphatic system, alleviate hydrocephalus, and improve neurological outcomes. The growing significance of mLVs makes targeted genetic manipulation essential for elucidating their biological roles.

To date, intra-cisterna magna (ICM) injection of the rAAV1 (or rAAV2/1) serotype has been the gold standard for mLV-specific gene delivery (10, 11). However, the predominance of rAAV2/1 has fostered an untested assumption that it is uniquely suited for mLVs targeting. No systematic comparisons of alternative rAAV serotypes have been conducted, leading to redundant serotype procurement and underutilization of existing tools. Here, we compare the infective performance of several commonly used rAAVs in CNS research on mLVs: rAAV2/1, rAAV2/5, rAAV2/8, rAAV2/9, rAAV2/BR1, and rAAV2/PHPeB. We found that all the tested rAAVs demonstrate robust infection in mLVs without any obvious dissemination into other organs or tissues which, most likely, can be attributed to the delivery route and doses itself. Importantly, our findings reveal that serotype choice is secondary to delivery strategy in determining mLVs tropism. This work establishes standardized protocols and expands the repertoire of viral tools for mLV-targeted research.

## 2 Materials and methods

### 2.1 Animals

Eight-week-old male C57BL/6 mice were purchased from Charles River Laboratories (CRL, Beijing, China). All animal protocols were approved by the Wenzhou Medical University (SYXK 2021-0020) and all the methods were performed accordingly, and in accordance with the Animals Research: Reporting of *In Vivo* Experiments (ARRIVE) guidelines and regulations. The mice were housed in groups of 4–5 in a climate-controlled facility, maintaining a temperature of 23 ± 3 °C and humidity of 50–60%. A 12-h light and 12-h dark cycle was implemented, and the mice had continuous access to water and food.

### 2.2 Tissue slice preparation and fluorescence detection

The mouse was deeply anesthetized with pentobarbital sodium, followed by transcardial perfusion using 30 mL of sterile phosphate buffered saline (PBS) solution and then 20 mL of 4% paraformaldehyde (PFA). The brain, spinal cord, cervical lymph nodes, heart, kidney, liver, and spleen were rapidly excised to minimize protein degradation. These tissues were then immersed in 4% PFA/PBS solution overnight for post-fixation, followed by 72 h in 30% sucrose phosphate buffer (PB) solution for dehydration. After dehydration, the tissues were embedded in optimal cutting temperature (OCT) compound and frozen on the sample table of a cryostat microtome. Sections (30 µm thick) of the striatum, lateral ventricle, hippocampus, cerebellum, cervical lymph nodes, heart, kidney, liver, spleen, and spinal cord were collected according to *The Mouse Brain in Stereotaxic Coordinates*. The tissue sections were mounted on slides and allowed to air-dry for at least four hours at room temperature before further experimentation. To remove any residual OCT compound, the sections were thoroughly washed three times with PBS solution for 10 min each. Circles around the tissue sections were outlined with a histochemical pen, and 4’, 6-diamidino-2-phenylindole (DAPI) solution was added. The slides were then incubated at room temperature for 10 min in the dark. Following incubation, excess DAPI solution was blotted away using filter paper, and the slides were coverslipped with anti-fade mounting medium to preserve fluorescence. Fluorescent signals from all tissue slices were captured using a confocal microscope, maintaining uniform exposure settings for all images to ensure consistent fluorescence intensity for comparative analysis. EGFP fluorescence quantification was conducted using ImageJ software (v1.53q, NIH). Fluorescence signals were isolated through the GFP channel segmentation function within ImageJ. Following background subtraction to eliminate nonspecific noise, a standardized auto-thresholding protocol (Otsu’s method) was applied to differentiate true signals from baseline noise. Regions of interest (ROIs) were manually delineated to match LYVE1-positive meningeal lymphatic vessels. Raw fluorescence intensity values were extracted from these ROIs for comparative analysis across experimental groups.

### 2.3 AAV production

The production of recombinant adeno-associated virus (AAV) involved plasmid preparation, AAV-HEK293T cell culture, transfection, viral purification, and titer quantification. First, the transgene of interest was cloned into an AAV vector plasmid (the complete AAV genome structure is illustrated in **Supplementary Figure 1**), which was co-transfected into AAV-HEK293T cells alongside the pHelper (providing adenoviral helper genes) and pAAV-RC (supplying AAV replication and capsid genes) plasmids using a transfection reagent in Opti-MEM medium. Cells were transfected at 70–80% confluency, followed by medium replacement with fresh complete medium after 6 hours. At 60 hours post-transfection, cells were harvested, lysed via freeze-thaw cycles, and treated with benzonase to digest residual nucleic acids. Cellular debris was removed by centrifugation, and the supernatant was precipitated with PEG8000/NaCl overnight. The viral particles were further purified through iodixanol gradient ultracentrifugation, with the 40% iodixanol fraction collected, dialyzed, and concentrated. Viral genomic titer was determined by quantitative PCR using alkaline lysate-treated samples and a plasmid standard curve.

### 2.4 Intra-cisterna magna injection

Mice were anesthetized via intraperitoneal injection of pentobarbital sodium (50 mg/kg) and secured in a stereotactic frame (Model 1900, RWD Life Science, Shenzhen, China). The mouse’s head was adjusted to an approximate 80° angle to achieve optimal vertical exposure of the cisterna manga, facilitating the subsequent injection process. After shaving and disinfecting the dorsal neck region with alternating applications of 75% ethanol and povidone-iodine, a midline skin incision was made to expose the occipital bone and underlying musculature. Superficial and deep neck muscles were gently separated via blunt dissection along the midline to visualize the atlanto occipital membrane and the cisterna magna. Various serotypes of rAAV were administered into the CSF-filled cisterna magna using a Hamilton syringe connected to an infusion pump (PHD Ultra, Harvard Apparatus, Holliston, MA, USA). Precision injection was maintained using a micro-syringe attached to glass electrodes under microscopic guidance (vector doses were described in **Table 1**). The rAAV vectors were diluted in sterile artificial CSF to a final volume of 10 μL and administered at a rate of 400 nL/min, resulting in a total injection duration of 25 minutes. Following the injection, the needle was kept in place for an additional 20 minutes to minimize leakage upon withdrawal. Medical bio adhesive glue was applied to seal the incision site on the dorsal neck to prevent potential virus leakage post-procedure. To support and expedite recovery, the mouse was placed on a 37 °C heating pad. Postoperative monitoring included close observation of the mice for activity, food intake, and mental status.

**Table 1:** The information of various viral vector.

Serotypes	Viral vectors (full name)	Titer (vg/ml)
rAAV2/1	rAAV-CMV-EGFP-WPRE-bGH-pA	5.53×10^12^
rAAV2/5	rAAV-CMV-EGFP-WPRE-bGH-pA	4.50×10^12^
rAAV2/8	rAAV-CMV-EGFP-WPRE-bGH-pA	4.81×10^12^
rAAV2/9	rAAV-CMV-EGFP-WPRE-bGH-pA	4.80×10^12^
rAAV2/1	rAAV-CAG-EGFP-WPRE-bGH-pA	5.01×10^12^
rAAV2/BR1	rAAV-CMV-EGFP-WPRE-bGH-pA	5.05×10^12^
rAAV2/PHPeB	rAAV-CMV-EGFP-WPRE-bGH-pA	5.09×10^12^

### 2.5 Intra-cerebroventricular injection

Mice were anesthetized via intraperitoneal administration of 1% pentobarbital sodium (50 mg/kg) and positioned on a thermostatically controlled heating pad. Cranial hair was shaved to expose the surgical field, followed by triple alternating disinfection with 75% ethanol and povidone-iodine. Ocular protection was achieved by applying erythromycin ophthalmic ointment to prevent corneal desiccation. Animals were securely fixed in a digital stereotaxic frame with ear bars. The skull surface was aligned to achieve horizontal zero plane (± 0.05 mm variance). Target coordinates for lateral ventricle injection were determined relative to bregma: anteroposterior (AP): -0.58 mm; mediolateral (ML): -1.10 mm; dorsoventral (DV): -2.20 mm. A microdrill was used to create a 0.5 mm craniotomy at the designated coordinates.

AAV2/9 viral vectors were loaded into a 10 μL Hamilton syringe mounted on a microprocessor-controlled infusion pump. Viral suspensions were delivered at 400 nL/min with a post-injection needle dwell time of 20 min to prevent backflow. The craniotomy site was sealed with adhesive. Viral expression was allowed to develop over a 14-day postoperative recovery period prior to experimental analysis.

### 2.6 Separation of meninges

The mouse was first perfused with ice-cold PBS, followed by 4% PFA administered through the left ventricle, ensuring careful puncture of the right auricle. Next, the back skin of the mouse was removed, and tissue scissors were meticulously used to sever the joint between the skull and cervical spine. Surgical scissors were then delicately employed to detach the skull from the foramen magnum along the superior border of the parietal bone, with gradual refinement of the area. During the insertion of the scissors into the foramen magnum, utmost care was taken to stay close to the bone to minimize any risk to the brain parenchyma, particularly the delicate cortex. The skull was then immersed in 4% PFA for 2 h for fixation. After fixation, the intact meninges were systematically dissected and removed from the interparietal, parietal, and frontal bones under microscopic guidance. Ophthalmic scissors were used to trim any bony protrusions around the skull, streamlining the retrieval process and reducing the risk of damage.

For whole-mount meninges staining, the meninges were blocked with a PBS solution containing 5% donkey serum, 1% Bovine Serum Albumin (BSA), and 0.1% Triton X-100 for 2 h at room temperature. They were then incubated overnight at room temperature with the primary antibody for LYVE-1 (1:200) diluted in PBS containing 1% BSA and 0.5% Triton X-100. Washing and DAPI staining procedures were performed similarly to brain section staining protocols. The meningeal tissue was carefully mounted onto a glass slide to ensure uniform placement and exposed to light for no more than 20 min to aid in proper drying. Given that the meninges are three-dimensional structures, unlike brain sections, efforts were made to flatten them as much as possible on the slide. If necessary, small incisions were made along the coronal suture to relieve tension and facilitate proper attachment.

### 2.7 Western blotting

The meningeal lymphatic vessels were first treated with RIPA buffer supplemented with phosphatase inhibitors and a phosphatase inhibitor cocktail. After lysis, the samples were homogenized and centrifuged at 10, 000 rpm for 10 min to remove cellular debris. Protein concentration was determined using the BCA assay kit. The samples were then denatured by heating at 90 °C for 8 min in 5X SDS loading buffer. Equal amounts of proteins were loaded onto either a 10% gradient polyacrylamide gel or an 8% gradient polyacrylamide gel for electrophoresis.

Following electrophoretic separation, the proteins were transferred onto a 0.45 µm PVDF membrane at 300 mA for 1h in an ice water bath. The membrane was then blocked using TBST buffer containing 1% BSA and incubated overnight at 4 °C with primary antibodies targeting β-actin (1:5000, 66009-1-Ig, Proteintech) and EGFP (1:1000, ab184601, Abcam). After primary antibody incubation, the membrane was treated with HRP-conjugated secondary antibodies (1:2500, 7074, Cell Signaling Technology) for 90 minutes at room temperature. Finally, protein bands were visualized using a molecular imager.

### 2.8 Statistical analysis

All datasets were analyzed using GraphPad Prism 9.0 and expressed as mean ± S.E.M. Normality was verified through Shapiro-Wilk testing (α=0.05). For two-group comparisons of normally distributed data, unpaired Student’s t-tests were applied; non-parametric Mann-Whitney U tests were used otherwise. Multi-group comparisons employed One-way ANOVA with Tukey’s *post hoc* analysis (parametric) or Kruskal-Wallis with Dunn’s correction (non-parametric), contingent upon normality and homogeneity of variance (Levene’s test, α=0.1). The correlation between viral load and EGFP fluorescence intensity was confirmed with Pearson correlation. Statistical significance was defined as *P* < 0.05.

## 3 Results

### 3.1 Targeted tropism of rAAV2/1 viral vectors for mLVs

**Figure 1:**
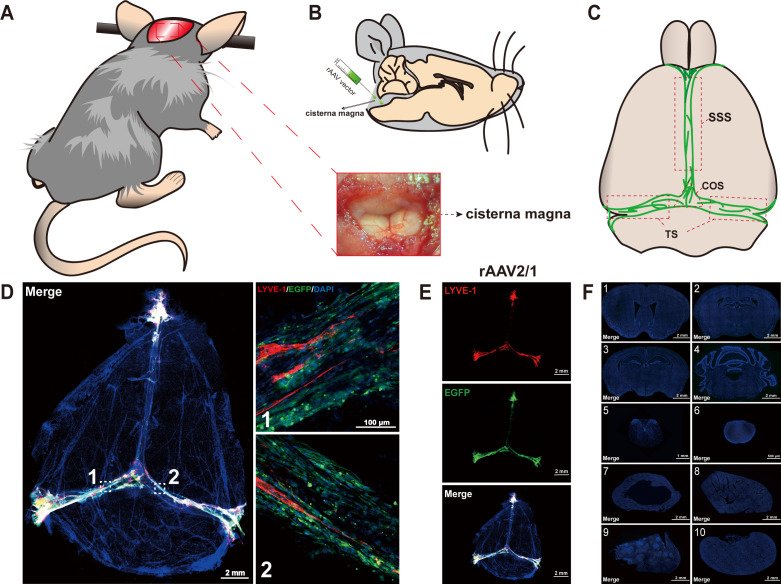
Intra-cisterna magna (ICM) injection procedure and representative images of C57BL/6 mice taken 2 weeks after ICM injection of rAAV2/1. **(A)** The mouse was immobilized on a stereotaxic frame, and a midline incision was made at the back of the neck. The image illustrates the fully exposed cisterna magna in the mouse. **(B)** The rAAVs were then injected into the CSF-filled cisterna magna. **(C)** The anatomical diagram provides details about the superior sagittal sinus (SSS), transverse sinus (TS), and confluence of sinuses (COS) of the dura mater. See comprehensive details of rAAVs in **Table 1**. **(D, E)** Two weeks post-ICM injection at 3×10^9^ vg/mouse, the rAAV2/1-CMV-EGFP-WPRE-pA fluorescence specifically localized to the mLVs tissue (n = 5 mice per group), revealing the SSS, TS, and COS of the dura mater within the mLVs. The merged images consist of the EGFP, LYVE-1 (marker for lymphatic vessels), and DAPI channels. **(F)** The rAAV2/1-CMV-EGFP-WPRE-pA fluorescence was scarcely detected in various brain slices and vital organs, including the corpus striatum (1), lateral ventricle (2), hippocampus (3), cerebellum (4), spinal cord (5), cervical lymph nodes (6), heart (7), liver (8), spleen (9), and kidney (10). The merged images consist of the EGFP and DAPI channels.

For efficient infection of mLVs, ICM injection of rAAV2/1 viral vectors was employed (**Figures 1A, B**). This method was used to directly expose the mLVs to the viral vectors, with an administered dose of 3×10^9^ vg/mouse. Subsequently, at 14 days post-injection, the mLVs, brain slices from different layers, and vital organs including cervical lymph nodes, heart, liver, spleen, etc., were harvested. Collected tissue slices were prepared and imaged following co-staining with a nuclear marker (DAPI), which facilitated the detection of signals from the fluorescent protein (EGFP). Additionally, LYVE-1 staining, a marker specific for mLVs, was performed to confirm precise infection of the mLVs. The results demonstrated that the fluorescence signals were robust and distinct, enabling the clear identification of the superior sagittal sinus (SSS), the transverse sinus (TS), and the confluence of sinuses (COS) of the dura mater within the mLVs after ICM injection of rAAV2/1 (**Figures 1C–E**). Moreover, the absence of significant infections in other tissues was verified (**Figure 1F**), indicating the exclusive infection capability of rAAV2/1 within the mLVs.

### 3.2 Comparative tropism of different rAAVs in mLVs

**Figure 2:**
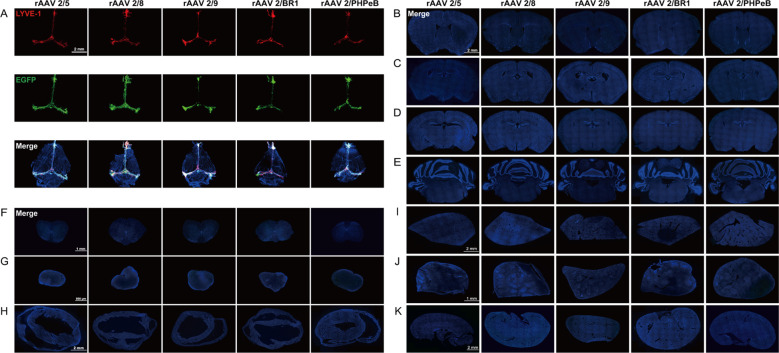
Representative images of C57BL/6 mice taken 2 weeks after ICM injection of various rAAV serotypes. **(A)** Following ICM injection with 3×10^9^ vg/mouse of rAAV2/5, rAAV2/8, rAAV2/9, rAAV2/BR1, and rAAV2/PHPeB, EGFP fluorescence was specifically localized to the mLVs tissue (n = 5 mice per group). The merged images consist of the EGFP, LYVE-1 (marker for lymphatic vessels), and DAPI channels. **(B-K)** Fluorescent images of the 5 rAAV serotypes showed minimal detectable fluorescent signals in brain slices and vital organs, including the corpus striatum **(B)**, lateral ventricle **(C)**, hippocampus **(D)**, cerebellum **(E)**, spinal cord **(F)**, cervical lymph nodes **(G)**, heart **(H)**, liver **(I)**, spleen **(J)**, and kidney **(K)**. The merged images consist of the EGFP and DAPI channels.

To evaluate the infective properties of various rAAV serotypes, five additional serotypes were generated: rAAV2/5, rAAV2/8, rAAV2/9, rAAV2/BR1, and rAAV2/PHPeB. Like the methodology employed for rAAV2/1, ICM administration (3×10^9^ vg/mouse) was utilized to adequately expose the mLVs to each viral vector. Fourteen days later, images of vital organs and whole brain scans for all the serotypes were provided. Notably, LYVE-1 staining revealed that EGFP signals were stably expressed in the mLVs across all rAAVs (**Figure 2A**). To assess potential infections in other brain regions, EGFP signals were examined, and none were detected in different brain regions (**Figures 2B–E**). Similarly, no EGFP signals were observed in the spinal cord (**Figure 2F**), cervical lymph nodes (**Figure 2G**), heart (**Figure 2H**), liver (**Figure 2I**), spleen (**Figure 2J**), or kidney (**Figure 2K**). The results conclusively demonstrated that all five serotypes of rAAV specifically infected the mLVs, with fluorescent signal scarcely detected in vital organs and various brain slices. In addition, no significant difference in EGFP signals was observed among the different serotypes, indicating the potential of alternative rAAV serotypes for exploring the function of mLVs.

### 3.3 mLV-specific infection depends on injection strategy (ICM injection)

**Figure 3:**
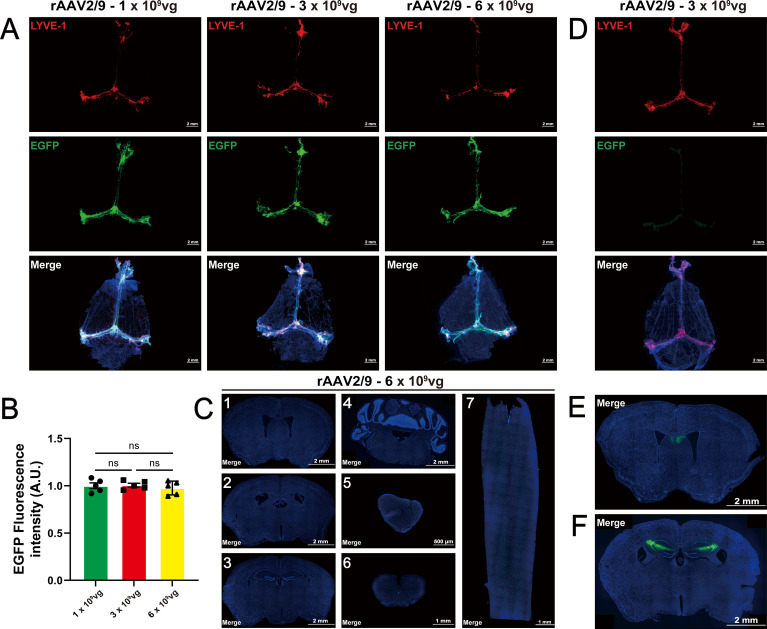
Potential rAAV expression across varying doses and injection modalities. **(A)** Mice received varying dose titers (1×10^9^ vg, 3×10^9^ vg, 6×10^9^ vg per mouse) of rAAV2/9 delivery in the mLVs and were perfused 2 weeks post-injection. Infection efficiency showed no significant difference among the different doses (n = 5 mice per group). The merged images consist of the EGFP, LYVE-1 (marker for lymphatic vessels), and DAPI channels. **(B)** Quantification of intrinsic EGFP fluorescence intensity in panel A (n = 5 mice per group), One-way ANOVA with Tukey’s multiple comparisons test. **(C)** No EGFP fluorescence signals were found following ICV administration of 6×10^9^ vg/mouse rAAV2/9 viral vectors in diverse brain slices and the spinal cord, including the corpus striatum (1), lateral ventricle (2), hippocampus (3), cerebellum (4), cervical lymph nodes (5), horizontal cervicothoracic spinal cord (6), and coronal cervicothoracic spinal cord (7). The merged images consist of the EGFP and DAPI channels. **(D)** Immunofluorescence images of mLVs infected with rAAV2/9 at 3×10^9^ vg/mouse following ICV injection for 2 weeks indicated subtle fluorescent signals (n = 5 mice per group). The merged images consist of the EGFP, LYVE-1 (marker for lymphatic vessels), and DAPI channels. **(E, F)** EGFP fluorescence signals were partially found at specific sites in the septal area **(E)** and hippocampus **(F)**. The merged images consist of the EGFP and DAPI channels. Unpaired t-test results: ns = no significance. Error bars represent the mean ± s.e.m.

It is well-known that rAAV2/9 serotype does not have specific infectivity and is widely expressed in the brain. However, our results indicated that ICM injection of rAAV2/9 led to specific infection in the mLVs. To further investigate, we performed ICM injections of rAAV2/9 at doses of 1, 3, and 6×10^9^ vg/mouse. Fourteen days post-injection, the images demonstrated that all volumes achieved mLV-specific infection (**Figure 3A**). Of note, at higher doses (1, 3, and 6×10^9^ vg/mouse), the transduction efficiency plateaued, showing no significant dose dependency (**Figure 3B**, One-way ANOVA, P = 0.5099 between groups), likely due to saturation of mLVs endothelial receptors. Consistent with our previous findings, no EGFP signals were even detected in other brain regions or tissues after ICM injection with 6×10^9^ vg/mouse rAAV2/9. Notably, no EGFP signals were observed in the sagittal and coronal images of spinal cords adjacent to the bulbar cisterna (**Figure 3C**). In contrast, ICV injection of rAAV2/9 (3×10^9^ vg/mouse) resulted in EGFP fluorescence in several regions, such as the septal area and hippocampus, but not in the mLVs (**Figures 3D–F**).

### 3.4 mLV-specific infection also depends on injected dosage (ICM injection)

**Figure 4:**
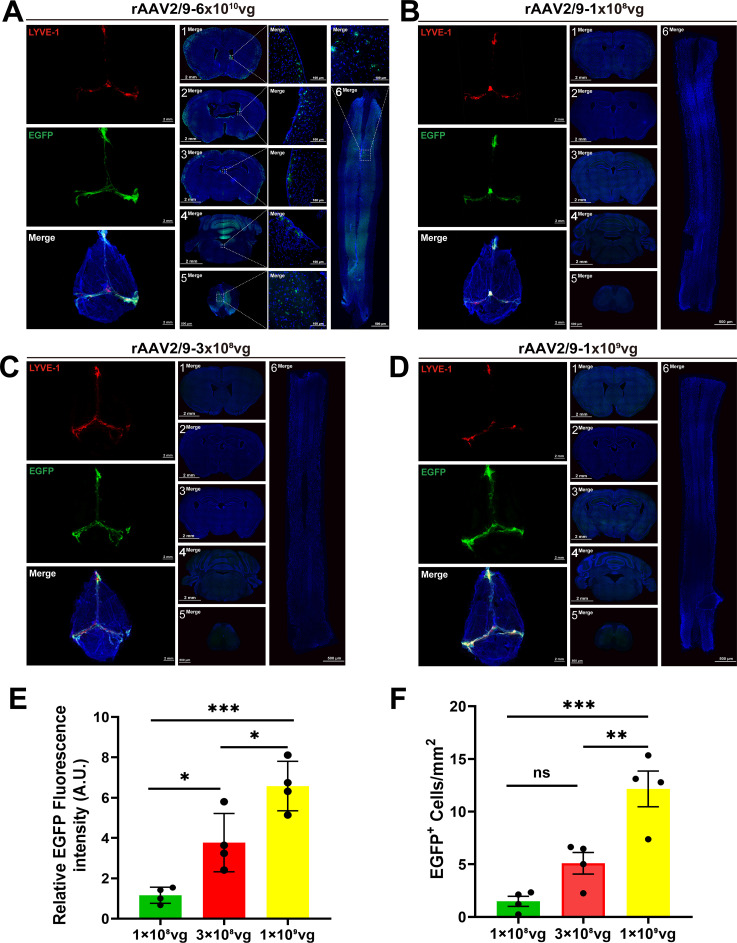
Dose response of AAV-mediated transduction efficiency in mLVs. **(A)** Two weeks post-ICM injection at 6x10^10^ vg/mouse, rAAV2/9-CMV-EGFP-WPRE-pA fluorescence was localized not only to mLVs but also revealed leakage across multiple brain regions and spinal cord. Fluorescence signals were detected in the striatum (1), lateral ventricle (2), hippocampus (3), cerebellum (4), horizontal cervicothoracic spinal cord (5), and coronal cervicothoracic spinal cord (6). The merged images consist of the EGFP, LYVE-1 (marker for lymphatic vessels), and DAPl channels. **(B-D)** Mice received escalating doses of rAAV2/9 (1x10^8^ vg, 3x10^8^ vg, 1x10^9^ vg per mouse) and were perfused at 2 weeks post-injection. EGFP fluorescence intensity exhibited a dose-dependent trend. No detectable EGFP fluorescence signals were observed in the striatum (1), lateral ventricle (2), hippocampus (3), cerebellum (4), horizontal cervicothoracic spinal cord (5), and coronal cervicothoracic spinal cord (6). The merged images consist of the EGFP, LYVE-1 (marker for lymphatic vessels), and DAPl channels. **(E)** Quantification of intrinsic EGFP fluorescence intensity among 3 groups with different dose concentrations (n = 4 mice per group), One-way ANOVA with Tukey’s multiple comparisons test. **(F)** Quantification of EGFP^+^ cells/mm^2^ among 3 groups with different dose concentrations (n = 4 mice per group), One-way ANOVA with Tukey’s multiple comparisons test: ns, no significance, ^*^P < 0.05, ^**^P < 0.01, ^***^P < 0.001. Error bars represent the mean ± s.e.m.

However, it is well known that AAV2/9 virus has a broad spectrum of infectivity on brain tissue, which appears to be inconsistent with our above results. We speculate that this might be related to the dosage of the virus injection. Thus, we administered ICM injection of AAV2/9 with different doses. As the data shown, although a high-dose virus (6×10^10^ vg/mouse) could still effectively infect the mLVs, this dose also led to infections in many other brain regions (such as striatum, lateral ventricle, hippocampus, cerebellum and spinal cords) (**Figure 4A**). By contrast, within the dosage range of 1×10^8^ vg/mouse to 1×10^9^ vg/mouse, the virus still exhibited specific infection of the mLVs (**Figures 4B–D**). Compared with the dosages of 1×10^8^ vg or 3×10^8^, the dosage of 1×10^9^ achieved more effective infection efficiency with higher EGFP fluorescence intensity and EGFP-positive cells (**Figures 4E, F**). However, exceeding this threshold may overwhelm local clearance mechanisms or saturate binding sites, leading to viral spread into adjacent CNS tissues as previously reported after cisterna magna infusion of substantially high-titer AAV (12, 13).

### 3.5 Long-term mLVs infection assessment with various rAAVs

**Figure 5:**
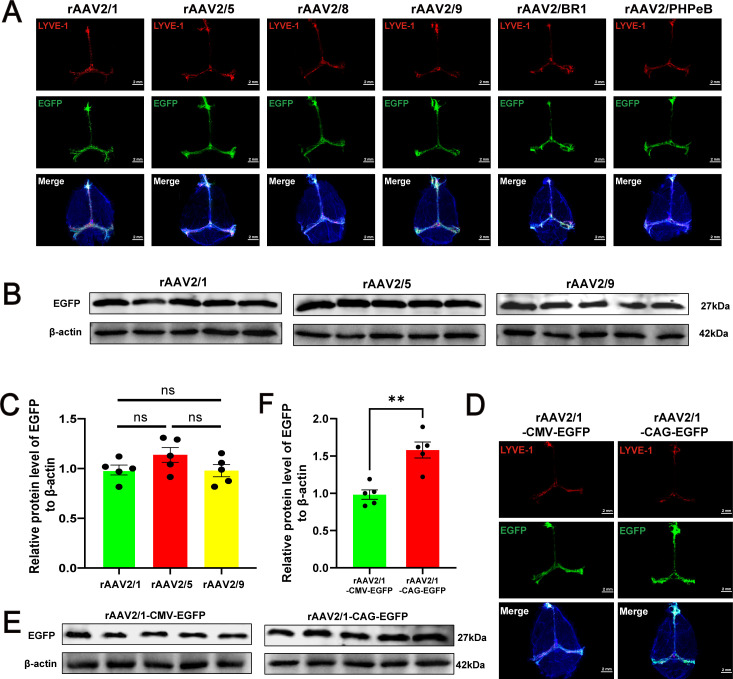
Timeline of rAAV infection and variations in infection efficiency due to different phenotypes and promoters. **(A)** Six months after ICM injection with 3×10^9^ vg/mouse, EGFP fluorescence specifically localized to the mLVs tissue (n = 5 mice per group). The merged images consist of the EGFP, LYVE-1 (marker for lymphatic vessels), and DAPI channels. **(B)** Western blot analysis of EGFP in mLVs of mice (n = 5 mice per group). Immunoblots were obtained from mLVs lysate of mice injected with rAAV2/1, rAAV2/5, or rAAV2/9 six months post-ICM injection. **(C)** Quantification of EGFP protein levels among the different rAAV serotypes (rAAV2/1, rAAV2/5, and rAAV2/9) (n = 5 mice per group), One-way ANOVA with Tukey’s multiple comparisons test. **(D)** Immunofluorescence images of EGFP in mLVs infected with rAAV2/1-CMV-EGFP or rAAV2/1-CAG-EGFP viral vector at 3×10^9^ vg/mouse, 2 weeks following ICM injection (n = 5 mice per group). The merged images consist of the EGFP, LYVE-1 (marker for lymphatic vessels), and DAPI channels. **(E)** Western blot analysis of EGFP in mLVs of mice (n = 5 mice per group). Immunoblots were obtained from mLVs lysate of mice injected with rAAV2/1-CMV-EGFP or rAAV2/1-CAG-EGFP two weeks post-ICM injection. **(F)** Quantification of EGFP protein levels between the rAAV2/1-CAG-EGFP group and the rAAV2/1-CMV-EGFP group (n = 5 mice per group). Unpaired t-test results: ns, no significance, ^**^P < 0.01. Error bars represent the mean ± s.e.m.

To assess the long-term persistence of these rAAVs, the aforementioned methodology was employed, and the intensity of fluorescence protein expression was measured 6 months post-administration. Discernible EGFP signals in the mLVs were observed, which were as vivid as those detected two weeks following the injection (**Figure 5A**). Western blotting confirmed that EGFP was stably expressed in the mLVs and showed no difference in mLVs infection performance among the different rAAV serotypes (rAAV2/1, rAAV2/5, and rAAV2/9) (**Figures 5B, C**). Additionally, we investigated whether different constitutive promoters affect the ability of rAAVs to infect mLVs, using rAAV2/1 as an example. Two weeks after the ICM injection of rAAV2/1-CMV-EGFP (3×10^9^ vg/mouse) or rAAV2/1-CAG-EGFP (3×10^9^ vg/mouse), mLVs were harvested for immunofluorescence analysis, revealing a notable difference in EGFP signal intensity between the two promoter groups (**Figure 5D**). WB analysis revealed that the protein abundance of EGFP was higher in the rAAV2/1-CAG-EGFP group compared to the rAAV2/1-CMV-EGFP group (**Figures 5E, F**). This suggests that the CAG promoter performs better than the CMV promoter in mLVs tissue. Overall, the data indicate enduring and stable transgene expression in the mLVs.

## 4 Discussion

Initially, a method is presented for the specific infection of mLVs through ICM injection of rAAV2/1. After administering 3×10^9^ vg/mouse of rAAV2/1 for a duration of 2 weeks, EGFP signals were detected in nearly all mLVs lining the entire dura mater. Observation of the EGFP fluorescence enabled the confident exclusion of potential expression of rAAV2/1 in other organs and tissues. Particularly in the cervical lymph nodes, which receive substances from the outflow of mLVs, no EGFP signals were observed. This finding aligns with a previous study indicating that rAAV2/1 delivered via ICM injection remains strictly localized to mLVs (5). Hence, the dosage of 3×10^9^ vg/mouse was deemed sufficient for mLVs infection.

Our study further revealed a critical dose-dependent dynamic in AAV-mediated mLVs transduction. While lower doses (1×10^8^–1×10^9^ vg/mouse) exhibited a strong positive correlation between viral load and EGFP fluorescence intensity, higher doses (≥3×10^9^ vg/mouse) showed no significant increase in transduction efficiency, suggesting saturation of AAV binding sites on mLVs endothelial cells. This saturation threshold (1×10^9^ vg/mouse) underscores the necessity of balancing viral load with target tissue capacity to avoid over-saturation artifacts. These findings emphasize that dose optimization is not merely a technical consideration but a mechanistic imperative for achieving specificity in mLV-targeted therapies.

Considering the injection strategy, ICM injection is currently deemed to be the most effective approach. While intraperitoneal (i.p.) or intravenous (i.v.) injections are more operationally convenient, it is important to note that the majority of rAAVs, including most of those evaluated in this study (rAAV2/1, rAAV2/5, rAAV2/8, rAAV2/9, and rAAV2/BR1), except rAAV2/PHPeB, encounter significant difficulty in penetrating the brain or possess limited ability to cross the blood-brain barrier (BBB), particularly since the expression of any given gene may be required in a particular CNS structure or cell type at a specific time during development (14–16). Furthermore, i.p. or i.v. injection can lead to widespread infection in peripheral tissues, rendering it unsuitable for precise manipulation of gene function in mLVs.

Moreover, it has been documented that ICV injection of rAAV2/9 carrying VEGF-C/VEGFR3 can induce lymphangiogenesis in mLVs in adult mice. Further, this administration strategy does not entirely eliminate the possibility of unintended infection in other brain regions (17, 18). ICV delivery requires invasive neurosurgery, which carries inherent risks of hemorrhage, CSF leakage, and direct parenchymal injury (18, 19). Numerous studies have demonstrated that ICV injection of the viral vectors can result in infection of the hippocampus, choroid plexus, cortex, and so forth (20–22). Therefore, there is a pressing need to develop new viral vectors variants specifically targeting mLVs for future clinical noninvasive strategies.

It is well established that rAAVs can persist in mammalian organs or tissues for extended periods without undergoing degradation by the cellular system as foreign DNA. Furthermore, they possess a minimal probability of integrating into the host genome. Owing to their low immunogenicity, rAAVs have emerged as promising tools for gene therapy in both animals and humans (23, 24). Different serotypes of rAAV exhibit distinct tissue tropism; notably, rAAV2/1 is commonly employed to infect the brain, muscle, retina, and vascular endothelial cells (22, 25, 26). Molecular characterization of mLVs confirms their classification as endothelial cells (5). Consequently, the utilization of rAAV2/1 in studying the functions of mLVs is based on its notable proficiency in infecting vascular endothelial cells. Expanding on this attribute, several rAAV serotypes with similar capabilities to rAAV2/1 were selected. Among these, rAAV2/5, rAAV2/8, rAAV2/9, rAAV2/BR1, and rAAV2/PHPeB are widely employed in neuroscience. For instance, the rAAV2/9 serotype exhibits a stronger tropism for retinal vascular endothelial cells (27). RAAV2/8 treatment could inhibit hepatic stellate cell activation and angiogenesis in mice with liver fibrosis (28). Indeed, our investigations revealed that all the rAAVs could achieve specific infection of mLVs without any discernible undesired infection in other areas. Similarly, it has been reported that ICM injection of rAAV2/9 was utilized to explore the role of mLVs in stroke (29). Hence, previous studies, in conjunction with the data presented here, corroborate that the majority of rAAVs used in brain infection are viable for functional studies of mLVs.

Regarding the specificity of mLVs targeting, LYVE1 is a well-established selective marker of lymphatic endothelial cells that shows minimal co-localization with vascular endothelial markers (e.g., CD31) or erythrocytes (30, 31). In our study, the partial overlap between AAV-delivered EGFP and LYVE1 signals aligns with prior AAV-based mLVs studies (32), demonstrating similar incomplete co-localization in meningeal preparations. While a minority of EGFP^+^ cells lacked LYVE1 co-labeling, the predominant tubular morphology of EGFP^+^ structures strongly supports mLV-specific targeting. We acknowledge that minimal off-target transduction of adjacent meningeal fibroblasts or immune cells may occur given the tissue’s cellular complexity; however, such events represent a small fraction of transduced cells and do not diminish our central conclusion of AAV9’s mLVs tropism.

Notably, our study revealed two unexpected findings requiring further interpretation: First, the lack of transduction efficacy differences across tested AAV serotypes despite their distinct cellular entry mechanisms; Second, the absence of dose-dependent transgene expression in mLVs (**Figure 3**), which contrasts with established AAV pharmacokinetics in peripheral tissues. We propose three non-exclusive hypotheses:

(1) Saturation of cellular uptake mechanisms at relatively high viral titers (≥1×10^9^ vg/mouse) may diminish dose-response correlations, as observed in AAV-saturated models (33, 34);

(2) This phenomenon likely arises from the dual determinants of AAV biology:

① Intrinsic serotype tropism: Natural receptor preference dictates baseline tissue specificity (e.g., AAV9’s galactose-binding capsid enabling BBB penetration (35); the observed pan-serotype mLVs specificity in our study implies an exceptionally high innate tropism of AAVs for mLVs endothelia.
② Delivery route reprogramming: Administration pathways can override native tropism by altering biodistribution. Intravenous AAVs are subjected to first-pass pulmonary sequestration, whereas localized ICM injection exploits the unique endothelial receptor landscape of mLVs to achieve route-specific targeting.

In our paradigm, ICM delivery synergizes with endogenous AAV tropism: While diverse serotypes inherently differ in systemic distribution (e.g., AAV9’s neurotropism vs. AAV5’s epithelial preference (36, 37), their forced localization to the CSF-mLVs interface through ICM injection creates a “funnel effect”. This spatially confines all serotypes to interact with the high-affinity receptor clusters on mLVs endothelia, effectively masking their native tropism differences. The unique CSF-lymphatic interface might exhibit high serotype-specific tropism for mLVs endothelial cells, overriding conventional dose dependency - a phenomenon recently reported in specialized BBB-penetrant AAV variants (38);

(3) The exceptionally high tissue specificity of AAVs for mLVs, mediated by their unique endothelial receptor profiles, might enable dose-independent transduction once minimal threshold binding is achieved (39).

In this study, through ICM injection, multiple serotypes of rAAVs successfully achieved precise and targeted transduction of mLVs without spreading to the brain parenchyma or peripheral organs. Notably, when the viral dose exceeded 1×10^9^ vg per animal, transduction efficiency did not exhibit a dose-dependent increase, suggesting potential saturation of receptor availability on mLVs endothelial cells.

While the ICM injection is an effective method for CNS-targeted drug delivery, this approach also carries inherent risks of CNS damage. The procedure requires direct puncture of the cisterna magna, which is located near the brainstem and critical vasculature, and thus poses a potential risk of medullary injury (40). Additionally, in a preclinical investigation of non-human primate tissues (NHPTs), AAV9 delivered via the ICM administration triggered immune responses by transducing and activating glial cells, including astrocytes and microglia. These responses elevated major histocompatibility complex (MHC) class II expression and promoted T cell infiltration. Pathological examination further demonstrated substantial necrosis and vessel infiltration within the striatum (41). Therefore, the ICM injection is technically challenging, which limits its clinical translation. However, compared with intravenous injection, ICM administration directly delivers the viral vector into the cerebellomedullary cistern. This approach reduces off-target effects while avoiding the hepatotoxicity risk associated with systemic delivery. Moreover, ICM injection bypasses the BBB, enabling direct access to the cerebrospinal fluid circulation. When compared to ICV and intrathecal (IT) injections, ICM administration is less invasive, minimizes parenchymal injury and secondary neuroinflammation, and reduces nonspecific viral diffusion. Additionally, studies have demonstrated that ICM delivery of adeno-associated virus encoding vascular endothelial growth factor C (AAV-VEGF-C) enhances meningeal lymphatic drainage by promoting lymphangiogenesis and functional improvement. This intervention attenuates microglial activation and neuroinflammation and improves cognitive outcomes (42). In summary, further in-depth research is still required in the future to validate the safety and efficacy of the ICM injection method.

The methodology established in this study enables efficient, durable, and targeted transduction of mLVs, thereby offering novel therapeutic avenues for diseases associated with mLVs dysfunction, such as Alzheimer’s disease (AD), stroke, and neuroinflammatory disorders. AD is a prevalent neurodegenerative condition with currently limited treatment options. Animal studies have demonstrated that disruption of mLVs or deficiency in AQP4 reduces Aβ clearance by up to 65%, significantly exacerbating the accumulation of Aβ/tau pathology, and triggering microglial activation, astrocyte proliferation, and cognitive decline. Therefore, targeting waste-clearance pathways based on the mechanisms of mLVs dysfunction may represent a promising therapeutic strategy for AD (42, 43). Intrathecal administration of an adeno-associated virus expressing full-length mouse VEGF-C (AAV-mVEGF-C) enhances CSF drainage into the deep cervical lymph nodes (dCLNs) by promoting lymphatic growth and upregulating neuroprotective signaling pathways. In a mouse model of ischemic stroke, pretreatment with AAV-mVEGF-C reduced stroke-related damage, improved motor performance during the subacute phase, and was associated with attenuated microglia-mediated inflammation and enhanced BDNF signaling in brain cells (44). Moreover, studies in K14-VEGFR3-Ig (K14) mice, which lack a functional meningeal lymphatic system, revealed that MLVs deficiency correlates with elevated local levels of the proinflammatory cytokine MCP-1 in the meninges, leading to an altered balance between pro- and anti-inflammatory mediators (45). In summary, mLVs dysfunction is closely linked to the pathogenesis and progression of various CNS diseases, though the underlying mechanisms require further investigation. The establishment of targeted mLVs transduction in this study will substantially advance our understanding of disease mechanisms and accelerate the development of innovative treatment strategies.

While this study establishes the foundational framework for serotype-agnostic mLVs targeting at optimal doses, future work will expand dose-response profiling across all serotypes. This includes systematic evaluation of transduction rate in CNS parenchyma at suprathreshold doses (>3×10^9^ vg), where serotype-dependent tropism may emerge due to differential receptor saturation or immune clearance (13, 46).

Several limitations warrant mention. First, our evaluation focused on rAAV serotypes common in neuroscience; lentiviruses or adenoviruses may offer complementary advantages. Second, while six-month expression confirms medium-term stability, lifelong persistence remains unverified. Third, due to technical limitations (the inability to separate the complete basal meninges), we were unable to evaluate the specificity of the infection on the basal meninges. The infection characteristics of different serotypes on the basal meninges need to be further clarified in the future. Finally, clinical translation requires noninvasive delivery strategies to circumvent ICM’s surgical complexity.

## 5 Conclusion

This work provides a standardized protocol for mLV-targeted gene delivery and expands the toolkit of serotypes compatible with this methodology. These methodological improvements will facilitate functional investigations of mLVs in CNS pathologies while providing foundational insights for therapeutic development.

## Data Availability

The original contributions presented in the study are included in the article/**Supplementary Material**. Further inquiries can be directed to the corresponding author.

## Supplementary material

Supplementary Figure 1 The complete genome structure of AAV. AAV, adeno-associated virus.
