# Supplementary figures and images for: Serotype-specific tropism of adeno-associated viruses in dorsal meningeal lymphatic vessels via intra-cisterna magna delivery
Source: Front Immunol. 2026 Mar 16;17:1768041. doi: 10.3389/fimmu.2026.1768041 (PMC13033482; doi:10.3389/fimmu.2026.1768041)

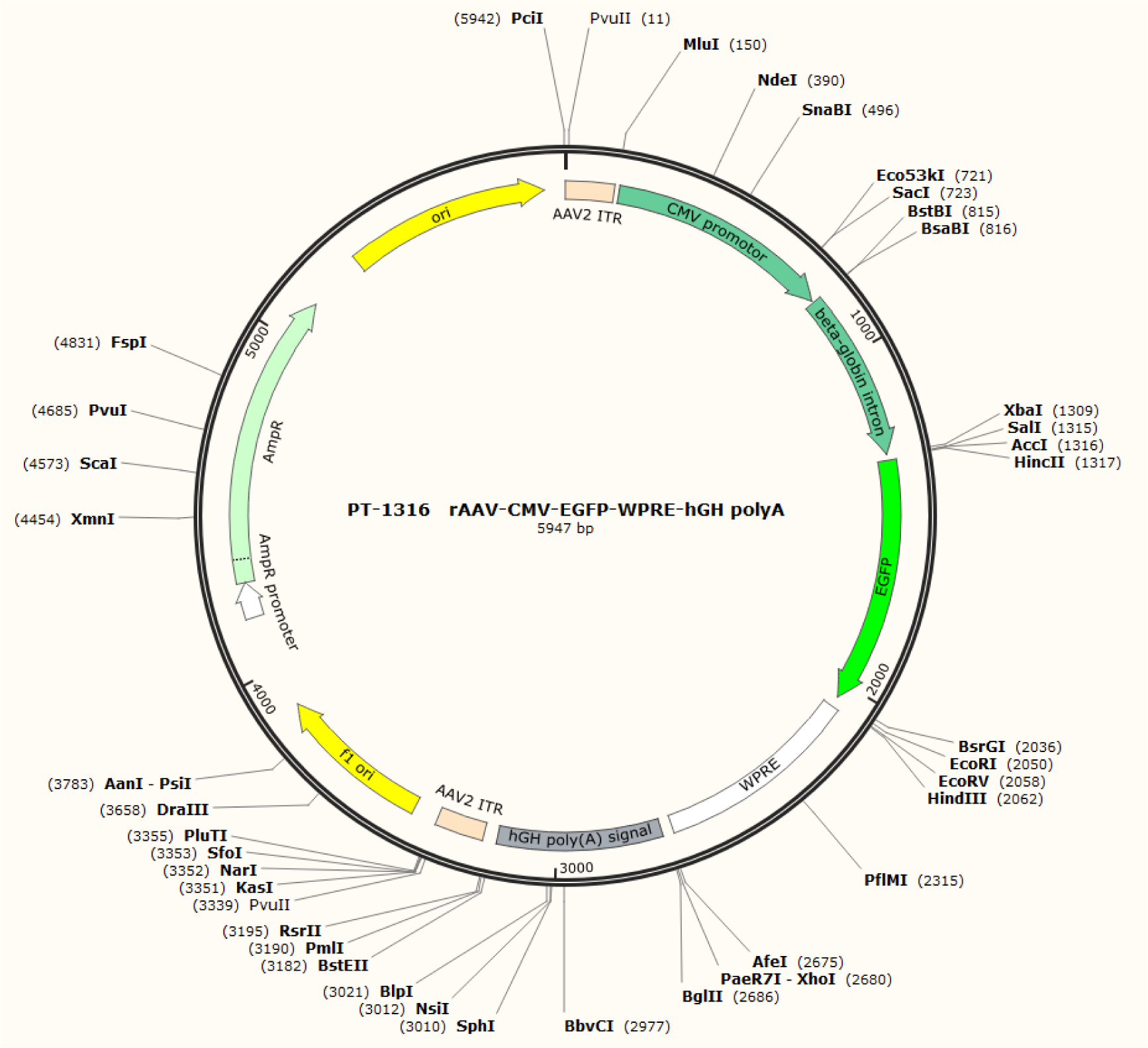

Supplement: Supplementary Figure 1 — The complete genome structure of AAV. AAV, adeno-associated virus. [file Image1.tif]
