# Supplementary material for: Serotype-specific tropism of adeno-associated viruses in dorsal meningeal lymphatic vessels via intra-cisterna magna delivery
Source: Front Immunol. 2026 Mar 16;17:1768041. doi: 10.3389/fimmu.2026.1768041 (PMC13033482; doi:10.3389/fimmu.2026.1768041)

The original uncropped images of western blot in Fig.5B

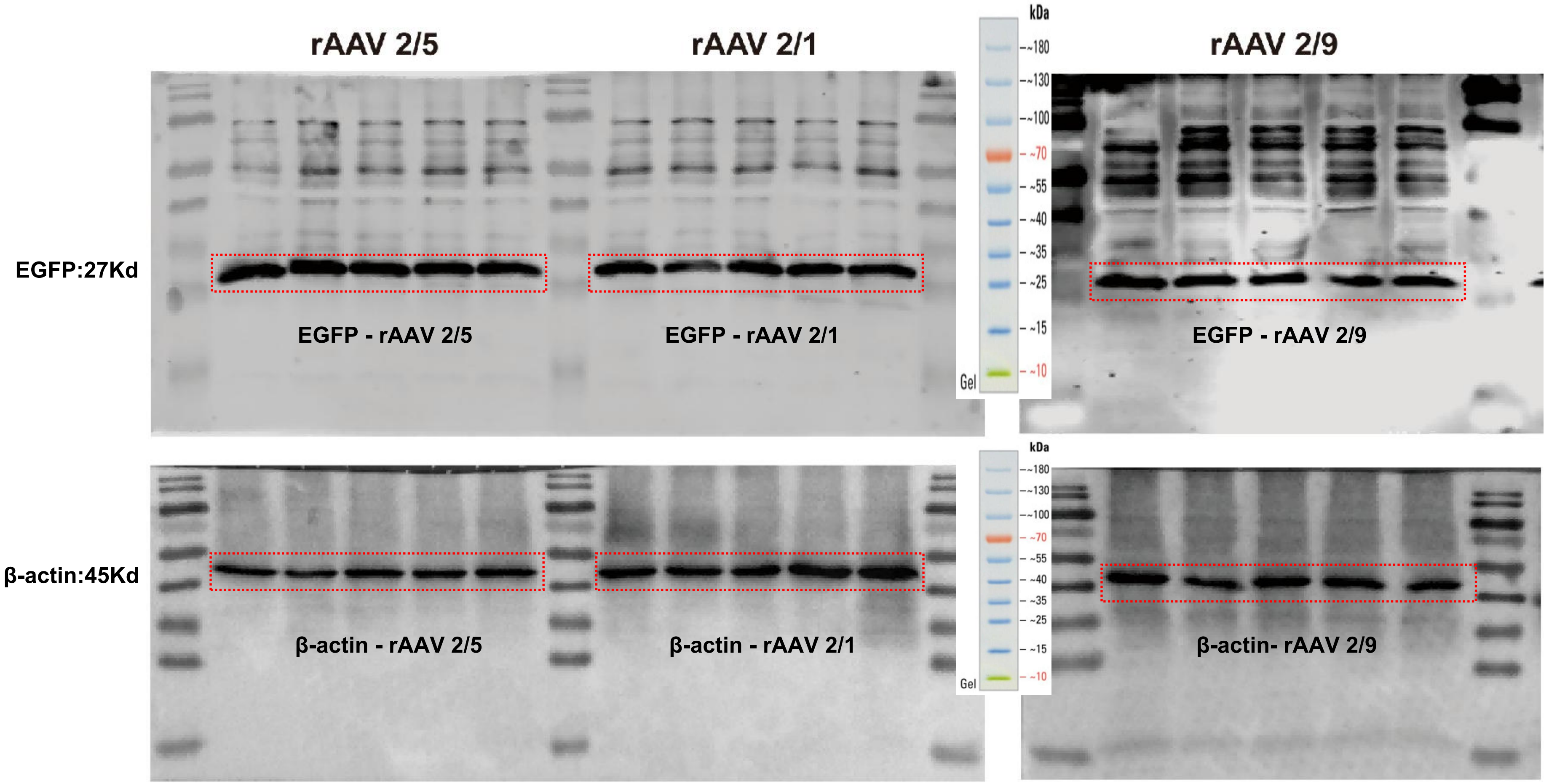

The original uncropped images of western blot in Fig.5E

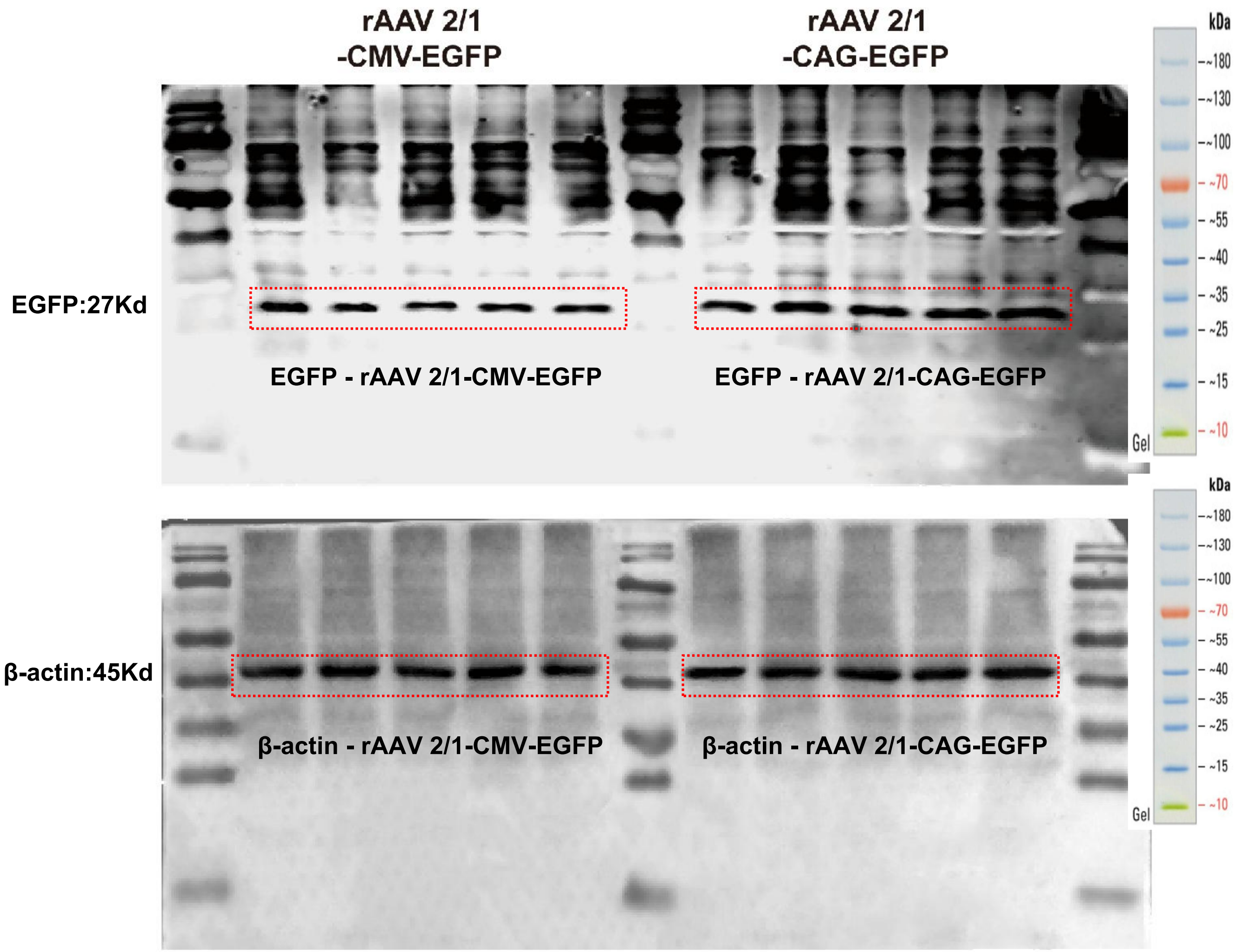

Supplement: Supplementary file 2 [file Supplementaryfile1.pdf]
